# Empirical research in medical ethics: How conceptual accounts on normative-empirical collaboration may improve research practice

**DOI:** 10.1186/1472-6939-13-5

**Published:** 2012-04-13

**Authors:** Sabine Salloch, Jan Schildmann, Jochen Vollmann

**Affiliations:** 1 Institute for Medical Ethics and History of Medicine, NRW-Junior Research Group "Medical ethics at the end of life: norm and empiricism", Ruhr University Bochum, Bochum, Germany

## Abstract

**Background:**

The methodology of medical ethics during the last few decades has shifted from a predominant use of normative-philosophical analyses to an increasing involvement of empirical methods. The articles which have been published in the course of this so-called 'empirical turn' can be divided into conceptual accounts of empirical-normative collaboration and studies which use socio-empirical methods to investigate ethically relevant issues in concrete social contexts.

**Discussion:**

A considered reference to normative research questions can be expected from good quality empirical research in medical ethics. However, a significant proportion of empirical studies currently published in medical ethics lacks such linkage between the empirical research and the normative analysis. In the first part of this paper, we will outline two typical shortcomings of empirical studies in medical ethics with regard to a link between normative questions and empirical data: (1) The complete lack of normative analysis, and (2) cryptonormativity and a missing account with regard to the relationship between 'is' and 'ought' statements. Subsequently, two selected concepts of empirical-normative collaboration will be presented and how these concepts may contribute to improve the linkage between normative and empirical aspects of empirical research in medical ethics will be demonstrated. Based on our analysis, as well as our own practical experience with empirical research in medical ethics, we conclude with a sketch of concrete suggestions for the conduct of empirical research in medical ethics.

**Summary:**

High quality empirical research in medical ethics is in need of a considered reference to normative analysis. In this paper, we demonstrate how conceptual approaches of empirical-normative collaboration can enhance empirical research in medical ethics with regard to the link between empirical research and normative analysis.

## Background

The methodology of medical ethics has shifted over the last two decades from a predominant use of normative-philosophical analyses to an increasing involvement of empirical methods. In the context of this so-called 'empirical turn' [1], a multitude of articles has been published in journals of medical ethics. The respective body of literature can be divided into two types of publications. The first type encompasses conceptual accounts of empirical ethics. Examples of this are publications which focus on the interplay between normative and empirical elements in empirical-ethical research [2-4], contributions on definitions of empirical ethics [5] or the various conceptual frameworks for empirical research in medical ethics [6-8]. A common feature of these publications is to conceptualise the ways in which empirical methods in combination with normative analysis can contribute to ethical research questions.

The second type of articles presents studies which use socio-empirical methods for research on concrete ethical issues. A broad range of topics has already been explored empirically in this way and the respective articles cover a wide spectrum in relation to their aims and the ways in which empirical research and normative analysis interact. Descriptive empirical studies in medical ethics restrict themselves to providing empirical knowledge on an ethical topic without further reference to or interaction with the normative debate. In contrast to these 'descriptive ethics studies', the work presented in other publications is based on a combination of empirical research and normative analysis. In these 'empirical-ethical studies', certain aspects of the empirical findings are linked to the ethical debate in order to demonstrate their contributions to normative reasoning. While the combination of normative reasoning and empirical research in some of these studies takes place against the background of specific concepts of empirical ethics [6-9], other studies do not aim at a *systematic *connection between their empirical data and the underlying normative questions (see, for example, [10,11]).

In this paper, we will argue that high quality empirical research in medical ethics is in need of a considered reference to normative research questions. Furthermore, we will defend the position that theoretical concepts of normative-empirical collaboration can enhance empirical research in medical ethics. To substantiate our claim, we will, firstly, present two typical shortcomings of empirical studies in medical ethics. We will then present and analyse two theoretical accounts of normative-empirical collaboration. In a further step, we will illustrate how these conceptual accounts can serve as remedies for the deficits of current practice of empirical research in medical ethics. Based on our analysis and our own experience with empirical research in medical ethics, we will conclude with a sketch of concrete criteria which may facilitate the planning and conducting of empirical studies in medical ethics.

## Discussion

### Shortcomings of empirical research in medical ethics

In a considerable number of the empirical studies which are currently published in journals of medical ethics or bioethics, the link between the empirical research and a normative analysis on the respective topic is not clear [12-14]. We would argue that publications on empirical studies in medical ethics as a normative discipline should always include at least some reference to normative analysis. Furthermore, we hold the view that an *explicit *connection between empirical data and normative reflection is a criterion for good quality empirical research in medical ethics. In the following, we will try to substantiate our claims with regard to good quality empirical research in medical ethics. In a first step, we will point out two deficits which concern the linkage between empirical and normative analysis and which can be encountered in the current practice of empirical research in medical ethics: (1) The complete lack of normative analysis in such research, and (2) cryptonormativity and a missing account with regard to the relationship between "is" and "ought" statements.

#### The lack of normative analysis: Purely descriptive studies in medical ethics

The first shortcoming of empirical studies in medical ethics concerns the complete lack of normative analysis or, to put it differently, the missing link between the empirical research and the ethical debate. A considerable number of the articles which are currently published in journals of medical ethics or bioethics present the results of empirical studies but remain on a descriptive level in their discussion and conclusion sections. One type of empirical research in medical ethics where these purely descriptive studies can particularly be found are quantitative surveys on stakeholders' attitudes regarding ethically challenging topics. An example of this type of research with regard to end-of-life decisions is the survey published by Craig et al. in 2007 [15]. This study examined the attitudes of a sample of 1,052 physicians towards physician-assisted suicide (PAS) in the US state of Vermont. One interesting finding of the empirical research is that physicians' opinions about the legalisation of PAS are polarised. Moreover, about half of the physicians indicated they would participate in PAS if it became legal in Vermont. In the end, the authors concluded: "Our findings contribute to a deeper understanding of some of the issues surrounding PAS. Specifically, we identified factors influencing physicians' opinions, and aspects of the PAS debate about which compromise is unlikely" [15], p. 403. In discussing their empirical findings, the authors remain on the descriptive level. However, there is no link between the study's findings and the normative issues relevant to the debate about an ethical justification of PAS.

Descriptive studies in medical ethics, such as the work of Craig et al., can be of excellent methodical quality measured against the background of the criteria established for socio-empirical research. In addition to their possible contributions to debates in social science such empirical studies can also be valuable for the field of medical ethics. This is the case, for example, if they deliver detailed and systematic analyses of stakeholders' moral experiences and attitudes and, therefore, contribute to a context-sensitive insight into certain moral practices in health care. Nevertheless, we would argue that the missing reflection on the impact of the empirical data for normative questions can be criticised as a shortcoming of empirical research from a medical ethics perspective. The most important reason for this position is that while the general need for empirical information in applied ethics is non-controversial, the specific kind of information which is required in argumentations about certain topics is dependent on the normative-ethical background which underlies the ethical evaluation [16,17]. As the specific need for empirical information is dependent on the underlying account of ethical justification, different kinds of empirical data on an ethical issue are not of the same use for a normative evaluation of the respective topic. Just to mention one quite obvious example: An ethical deliberation on a utilitarian basis will usually need other empirical information than an argumentation which is based on a Kantian view of morality. Hence, if empirical data are gathered without prior reflection about their significance within a normative deliberation on the respective topic, these data's relevance for ethics as a normative discipline must be regarded as more or less accidental. Therefore, a reflection on the presumably ethical significance of empirical data prior to the beginning of an empirical study would be desirable.

A considered reference to the normative side is also important in the case of empirical studies in medical ethics which aim at identifying new ethical challenges in practice which have not yet been recognized and discussed. The 'exploratory function' of empirical research is also dependent on normative-ethical presuppositions which decide if and in what sense the empirically identified issue can be seen as an *ethical *problem and not as a practical problem of another origin. The chosen background of ethical evaluation, furthermore, determines the kind of additional data which is needed to arrive at an ethical judgment about the respective issue. Therefore, we would argue that at least some conception of the relationship between the empirical research question and the normative debate on the respective topic should underlie empirical studies which want to contribute to medical ethics as a normative field.

#### Cryptonormativity and the is-ought problem

A second shortcoming frequently identified and criticised in empirical research in medical ethics rests upon the problem of drawing normative conclusions from empirical findings [3,4,16]. Studies which derive normative statements from their descriptive data alone run the risk of an is-ought fallacy by ignoring the fact that ethical values, norms and principles play an irreducible role in ethical judgment. Whereas it is of great importance for ethics to investigate the cultural, historical and psychological contexts of moral decision-making, this does not mean that empirically detected moral motives and behaviour are together ethically justified.

Studies which draw normative conclusions from empirical results often have a cryptonormative character, which means that they *implicitly *take normative statements as the basis of their ethical argumentation without mentioning or reflecting on them.

This drawback can frequently be found in publications of empirical studies which entail normative statements in their conclusion sections. Two types of unclear normative conclusions can be distinguished here. In the first case, normative statements are directly drawn from the empirical findings. In the second case, normative statements are found in the conclusion sections which are not explicitly linked to the results of the empirical study, but nevertheless, it can be asked from where these statements are derived. In both cases, the normative premise is not made explicit in the argument but is necessary to arrive at the normative conclusion.

One illustration of the second type of unclear relationship between empirical data and normative conclusions is a paper by Bendiane et al. which, in a similar way to the study of Craig mentioned above, deals with the issue of physician-assisted suicide [18]. In this study, French hospital nurses were asked whether euthanasia and PAS should be legalised for patients with incurable conditions. The study showed that 48% of the nurses supported the legalisation of euthanasia and 29% supported the legalisation of PAS. Furthermore, the authors showed that reported training in palliative care was negatively correlated with nurses' support for a legalisation of PAS. The authors concluded that: "Improving professional knowledge of palliative care would improve the management of end-of-life situations, but it could also help to clarify the debate over euthanasia" [18], p. 243.

While the demand for an improved training in palliative care as such is important, in the context of this study's empirical results, the authors' claim can be misleading. This is because, in our view, the normative statement (improvement of palliative care education) cannot be linked to the empirical findings (better education is associated with a decrease in the support of PAS). If such a link between the empirical results and the normative statement in the conclusion section is made, this can be problematic: For example, one may read the paper as if the authors do not make a hidden normative statement explicit when they plead for a better education in palliative care. Following the results of the empirical study, a better knowledge in palliative care might lead to a decrease in the support for legalisation of euthanasia and PAS in France. However, the question whether euthanasia and PAS should be legalised is itself a normative question which has strong ethical implications. Therefore, if the authors plead for better palliative training in the conclusion of *this *study, their statement can be understood as implicitly taking a stand against the legalisation of euthanasia and PAS in France. However, this ethical standpoint has not been discussed normatively in the study but is implicitly taken as a basis for argumentation.

After this characterization of two drawbacks in the current practice of empirical research in medical ethics, we will now present two theoretical conceptions of the normative-empirical relationship which may contribute to an improved practice of empirical research in medical ethics.

### Conceptual accounts of normative-empirical collaboration and their contributions to research practice

In recent years, a number of conceptual accounts regarding the normative-empirical collaboration in medical ethics have been published. In the following, we will present two of these conceptual frameworks which may be useful for researchers who are planning empirical studies in medical ethics and who aim at an integration of empirical research and normative analysis. Although both models presented may not be fully sufficient to provide concrete guidance for planning and conducting an empirical study, we appreciate both models as they acknowledge that a social practice can be judged by both the gathering of empirical data and normative ethical analysis. Furthermore, the two models conceptualize the interaction between both elements in a plausible and systematic way which may be the most important criterion for a good concept of empirical research in medical ethics.

Birnbacher's as well as Leget et al.'s model share the characteristic that they rest upon certain meta-ethical claims, such as a cognitivist view of ethics and the acknowledgement of the fact-value distinction. Hence, the models provide a suitable theoretical background for those researchers who are in accordance with these presuppositions. In contrast to other accounts, the models of Birnbacher and Leget have not been tested in empirical studies so far.

They can be distinguished from other models currently applied in medical ethics, such as hermeneutic ethics or reflective equilibrium, for example, [7-9], which provide alternative accounts of the normative-empirical relationship and different methodological strategies.

However, the two models on normative-empirical collaboration chosen differ in several important characteristics, such as their disciplinary background and their aims. The first model by Dieter Birnbacher, a German philosopher, provides a concept of the relationship between ethics as a theoretical discipline and morals as an empirical phenomenon [17,19]. He discusses different steps in the examination of concrete ethical problems from the perspective of an ethicist. In contrast to this, the second model by Leget and colleagues [20] draws on a categorisation of methods for integrating empirical research and normative ethics which has been developed in the context of the "empirical turn" [21]. Leget et al., more than Birnbacher, make reference to the interdisciplinary challenge of doing empirical research in bioethics. Although the two approaches have the aforementioned and other differences, we believe that both can be useful for researchers who aim to improve the integration of empirical research and normative deliberation in medical ethics. Both approaches will be presented in a short form, followed by a discussion of how they can be used as remedies for the two shortcomings of empirical research in medical ethics which have been discussed previously.

#### Birnbacher's four tasks of applied ethics

One of the first, but still vividly discussed, concepts of the collaboration between ethics and social sciences is that of Dieter Birnbacher [17,19]. Birnbacher displays a model of the interrelationship between empirical information and ethical thinking where he distinguishes four interdependent aspects of an ethical examination of empirical moral phenomena. He firstly describes the *analysis *part, which consists of a clarification and reconstruction of moral concepts, arguments and ways of reasoning [19], p. 45. The next step, called *critique*, is a critical assessment of concepts and explanatory statements used in a certain moral context to arrive at clarity, unambiguousness and plausibility. *Construction*, which follows, means the development of an ethical approach and evaluation of the moral issue at stake; for instance, a construction of ethical norms that are specific to this particular context. The last aspect Birnbacher mentions is *moral pragmatics*, which is concerned with the practical, political or educational, implementation of moral norms, assuming that it is not only sufficient for an ethicist to discuss the moral rightness or wrongness of a certain practice on a theoretical level, but also to think about the conditions under which a moral norm or value can become effective in society.

According to Birnbacher, the construction and the pragmatic part are particularly dependent on empirical information and, therefore, on interdisciplinary cooperation. While in the construction part, empirical data are necessary for the development of context-specific moral norms, in the implementation phase, knowledge from empirical disciplines is needed to effectively influence people's attitudes and behaviour. Nevertheless, Birnbacher's position can be completed in pointing out that the first two tasks of ethics which he describes ("analysis" and "critique") similarly rely on empirical cooperation: For a clarification and reconstruction of moral arguments, empirical knowledge about the arguments which are employed in a certain context is also very important, as empirical data are necessary for a critical examination of the truth of certain claims on which ethical argumentations are based.

Birnbacher's general account of empirical-normative collaboration can be applied to empirical studies in medical ethics. In general, it may be helpful for scientists conducting empirical studies in medical ethics to think about where to position their scientific work within this model of ethical reasoning. This positioning has an influence on the kind of information which is needed for a normative discussion of the respective issues. If researchers, for instance, want to contribute to the analysis part, other empirical studies may be more useful than if they want to be conducive on the level of moral pragmatics. In helping to clarify the significance of empirically derived knowledge in specific ethical deliberations, Birnbacher's approach may provide support for those empirical studies in medical ethics which suffer from a lack of normative analysis. Here it can be said, just to mention one example, that an empirical study can contribute on the level of moral pragmatics. The empirical results of a study on people's moral attitudes may provide policy makers with relevant information about chances and challenges if the regulation of an ethically relevant issue was to be changed. Knowing beforehand that the public is highly polarised regarding this issue, for example, may enable policy makers to think about appropriate provisions before the implementation of a new law. In general, Birnbacher's model may help to make clear in which part of an ethical deliberation empirical data are to be integrated.

#### Leget et al.'s 'critical applied ethics'

A second conceptual approach on empirical-normative collaboration was presented by Leget et al. in 2009 under the title of 'Critical Applied Ethics' [20]. The authors describe a close interdisciplinary collaboration between ethicists and social scientists. Normative and empirical aspects are seen as two independent foci on one bioethical 'ellipse', which means that both perspectives are kept distinguished, but that they, nevertheless, influence each other in a fruitful way [20], p. 231. Normative and empirical disciplines investigate the same social practice using their respective methods during the five stages of the research process which are described by the authors: Determination of the problem, description of the problem, effects and alternatives, normative weighing, and evaluation of the effects of a decision. At the level of problem description, for example, possible empirical contributions can encompass the careful study of people's motives, actions and intentions by the social scientist, while the ethicist's task consists of a critical look at the concepts and vocabulary used in this specific context. At the point of "normative weighing", which forms a later stage of the research process, normative theory renders moral judgment, while the descriptive sciences' task is a critical examination of the ethical theories brought into play and the detection of possible empirical (for example, anthropological) premises within them. Thus, the strong interdisciplinary collaboration allows for a mutually critical look at each other's discipline and its premises and presuppositions, as well as at the social practice which is examined and criticized.

This model of interdisciplinary cooperation, as described by Leget et al., can be very useful for researchers in medical ethics, as it provides a systematic account of the different stages of an empirical study in medical ethics. Furthermore, this model can help the representatives of the empirical, as well as of the normative sciences, to become aware of and explicit about the different roles they fulfil as the research process progresses. Along these lines, the concept of 'Critical Applied Ethics' may also lead to a clearer distinction between normative and descriptive statements in the publications of empirical studies. It can also it can help to avoid unclear normative statements as conclusions from empirical data in discussing openly the normative presuppositions which underlie the research project and in reflecting on them critically up to the point of data interpretation.

### Conceptual and practical aspects of empirical-normative collaboration - further perspectives

The preceding analysis illustrates how conceptual accounts of empirical-normative collaboration may contribute to the practice of empirical research in medical ethics. Reference to the existing models can stimulate a reflection on how to combine empirical research and normative analysis in a systematic way. While the focus of our paper is to analyse the contribution of conceptual accounts of normative-empirical collaboration to empirical research, it should be noted that the practice of empirical research in medical ethics may also be of value for the conceptual accounts of empirical-normative collaboration in medical ethics. Only a few of these concepts have so far provided the basis for concrete empirical-ethical studies, such as approaches which are based on a reflective equilibrium [9], a symbiotic model of empirical ethics [6] or a hermeneutic account [7]. At the same time, there are many other concepts which, to our knowledge, have not yet been empirically 'tested' in this sense, such as the approaches of Birnbacher [19] and Leget et al. [20], which have been presented previously. Nevertheless, conducting an empirical study may offer the opportunity to check the practicability of conceptual approaches and can lead to their refinement or modification. Such a modification of a conceptual account could, for example, necessitate a redevelopment of the different stages which are described in the theoretical model. Another reason for modification may be triggered by the fact that research practice reveals problems in interdisciplinary communication or cooperation which are not considered in the theoretical model but should, nevertheless, be integrated.

Our analysis also sheds light on the current discourse about quality criteria for empirical research in medical ethics [22,23]. As outlined in the introductory part, the premise of our article is that the development and analysis of empirical work in medical ethics should take place with reference to the relevant normative debate(s). Based on this assumption, we have defended the thesis that the quality of empirical studies in medical ethics can be enhanced by a closer connection between empirical research and theoretical approaches to the normative-empirical collaboration in medical ethics. Nevertheless, we acknowledge that our view of normative analysis as a core feature of research in medical ethics needs further specification to determine which quality criteria should be applied for empirical studies in medical ethics. This is true with respect to the threshold of what amount and type of normative analysis should be expected from these studies. While it is outside the scope of this paper to elaborate further on this question, it should be noted that, depending on the respective conception of empirical-normative collaboration, there will be different criteria for good quality in empirical-ethical research. In any case, we expect that, to some degree, such research has to meet the basic standards of both empirical and normative methods. Over and above quality criteria for the empirical research which is done (for example, "Have the empirical methods been applied appropriately?" or "Are the results presented in a clear and transparent way?"), standards should be formulated which bear on the articulation between normative and empirical aspects [23]. The development of quality criteria for empirical studies in medical ethics should take into account the challenges which arise from the need for an integration of empirical research findings and normative analysis which is specific for these studies.

Not least because of these aspects, empirical research in medical ethics is an especially challenging form of interdisciplinary research. However, a number of the already existing theoretical accounts of normative-empirical collaboration do not provide researchers with information which is concrete enough to set up an empirical study in medical ethics on their basis alone. Based on our analysis in this article, as well as our own practical experience with doing empirical research in medical ethics [24-26], we suggest the following concrete steps when considering an empirical study on a specific topic.

1) Empirical and normative research questions should be formulated in a careful way before starting empirical research in medical ethics. At the same time, it should be considered how these research questions are interrelated, for example, if and how the answer to the empirical question is necessary to answer the normative research question. Furthermore, the identification of possible biases is a crucial point: Normative interests can lead to bias in the interpretation of the empirical data, and the state of empirical research may lead to a bias in the formulation of the normative question.

2) The conceptual and effective interplay between normative and empirical aspects should be considered from the beginning of an empirical study, and this reflection should continue up to the point of data interpretation and publication of the results. This also means that a mutually critical view of the disciplines involved is desirable during the whole research process. This mutually critical reflection may concentrate on implicit normative or empirical premises, as well as on underlying assumptions of theories and methods which are applied in the research project.

3) Empirical research in medical ethics should take place in the form of an ongoing, open and constructive cooperation between representatives of the normative and the empirical sciences. This means that the participating researchers should be open to critique and re-adjustment of their own positions, and acknowledge that there are different perspectives on the same topic which should be integrated to arrive at an empirically informed ethical judgment.

4) The results of empirical studies in medical ethics should be presented in a clear and transparent way which is compatible with the basic standards of the disciplines involved. In addition, the development of new forms of publication of empirical-ethical studies would be preferable (for example, an adaptation of journal standards) which account for the specific demands of this form of interdisciplinary research.

We do not intend to display a new model for normative-empirical collaboration here. Possibly, the implementation of already existing theoretical conceptions into the research practice of empirical medical ethics may be even more desirable at this point than an extension of the spectrum of different approaches to normative-empirical collaboration. In allowing for a reflection of the interaction between normative and empirical elements in ethical deliberation, empirical research in medical ethics can become a very fruitful enterprise and can aid the treatment of the complex ethical challenges of modern health care.

## Summary

A considered reference to normative research questions can be expected from good quality empirical research in medical ethics. In this paper, we have defended the thesis that the quality of empirical research in medical ethics can be enhanced by taking into account conceptual accounts of the normative-empirical relationship. Overcoming the missing connection between theory development and research practice in empirical medical ethics may also prove profitable for the theoretical concepts of empirical-normative cooperation. Our research further suggests that the discussion on quality criteria for empirical studies in medical ethics should take into account the specific challenges which arise from the need to bring together normative and empirical aspects in this interdisciplinary research field. We concluded with some further suggestions regarding the research practice of empirical studies in medical ethics.

## Competing interests

The authors declare that they have no competing interests.

## Authors' contributions

All authors have contributed substantially to the conception and design of the manuscript. SS and JS have drafted the manuscript. They contributed equally. JV has critically revised the manuscript. All authors have read and approved the final manuscript.

## Funding

This publication is a result of the work of the NRW Junior Research Group "Medical Ethics at the End of Life: Norm and Empiricism" at the Institute for Medical Ethics and History of Medicine, Ruhr-University Bochum which is funded by the Ministry for Innovation, Science and Research of the German state of North Rhine-Westphalia.

## Pre-publication history

The pre-publication history for this paper can be accessed here:

http://www.biomedcentral.com/1472-6939/13/5/prepub
